# Incident Colorectal Cancer in Inflammatory Bowel Disease

**DOI:** 10.3390/cancers14030721

**Published:** 2022-01-30

**Authors:** Benedetto Neri, Maria Lia Scribano, Alessandro Armuzzi, Fabiana Castiglione, Renata D’Incà, Ambrogio Orlando, Stefano Festa, Gabriele Riegler, Walter Fries, Gianmichele Meucci, Patrizia Alvisi, Filippo Mocciaro, Claudio Papi, Michelangela Mossa, Giorgia Sena, Luisa Guidi, Anna Testa, Sara Renna, Iris Frankovic, Anna Viola, Marta Patturelli, Carlo Chiaramonte, Livia Biancone, on behalf of IG-IBD (Italian Group for the Study of Inflammatory Bowel Disease)

**Affiliations:** 1 GI Unit, Department of Systems Medicine, University of Rome “Tor Vergata”, 00133 Rome, Italy; benedettoneri@gmail.com (B.N.); michelangela.mossa@ptvonline.it (M.M.); giorgia.sena@ptvonline.it (G.S.); 2 Gastroenterology Unit, AO San Camillo Forlanini, 00152 Rome, Italy; marialiascribano@virgilio.it; 3 IBD Unit, Department of Biomedical Sciences, IRCCS Humanitas Research Hospital, 20089 Rozzano, Italy; alearmuzzi@yahoo.com; 4 Gastroenterology, Department of Clinical Medicine and Surgery, Università Federico II, 80131 Naples, Italy; fabcasti@unina.it (F.C.); annatesta82@virgilio.it (A.T.); 5 IBD Unit, Gastroenterology, Azienda-Università of Padova, 35121 Padua, Italy; dinca@unipd.it; 6 IBD Unit, “Villa Sofia-Cervello” Hospital, 90146 Palermo, Italy; ambrogiorlando@gmail.com (A.O.); sara.renna.md@gmail.com (S.R.); iris.frankovic.1@studenti.unipd.it (I.F.); 7 IBD Unit, S. Filippo Neri Hospital, 00135 Rome, Italy; tefano.festa@aslroma1.it (S.F.); claudio.papi@aslroma1.it (C.P.); 8 Department Precision Medicine, Università degli Studi della Campania Luigi Vanvitelli, 81100 Caserta, Italy; gabriele.riegler@unicampania.it (G.R.); m.patturelli@studenti.unina.it (M.P.); 9 IBD Unit, Department of Clinical and Experimental Medicine, University of Messina, 98122 Messina, Italy; fwalter@unime.it (W.F.); aviola@unime.it (A.V.); 10 Gastroenterology Unit, San Giuseppe Hospital, 52100 Arezzo, Italy; g.meucci@teletu.it; 11 Gastroenterology Unit, AUSL Bologna, 40133 Bologna, Italy; patrizia.alvisi@ausl.bologna.it; 12 Gastroenterology and Endoscopy Unit, ARNAS Civico Di Cristina-Benfratelli, 90127 Palermo, Italy; filippo.mocciaro@arnascivico.it; 13 IBD Center, Fondazione Policlinico A. Gemelli IRCCS, Università Cattolica del Sacro Cuore, 00168 Rome, Italy; luisa.guidi@policlinicogemelli.it; 14 Statitician, Department of Biomedicine and Prevention, University of Rome “Tor Vergata”, 00133 Rome, Italy; Chiaramonte@med.uniroma2.it

**Keywords:** Inflammatory Bowel Disease, colorectal cancer, incident cancer, clinical outcome

## Abstract

**Simple Summary:**

The sequence chronic inflammation-dysplasia-cancer is involved in the development of several gastrointestinal cancers, including colorectal cancer (CRC) in Inflammatory Bowel Disease (IBD). Several risk factors for CRC are recognized in Ulcerative Colitis (UC) and Crohn’s Disease (CD) colitis. The combined role of IBD characteristics and incident CRC, including CRC-related symptoms at onset, in determining the long-term outcome needs further investigation. These issues were addressed in our multicenter study in IBD patients with incident CRC. CRC was more frequently diagnosed by colonoscopy in UC and by imaging in CD. CRC occurred in one fourth of patients at a young age (≤40 years), and a high rate of CRC-related mortality was observed, particularly in patients with CRC diagnosed at an older age. The present findings from incident CRC in IBD support the need to focus the attention on surveillance programs in subgroups of patients at higher risk for CRC and CRC-related death.

**Abstract:**

Colorectal cancer (CRC) risk is increased in Inflammatory Bowel Disease (IBD) and surveillance needs to be tailored according to individual risk. The open issues include the role of the characteristics of IBD and CRC in determining the long-term outcome. These issues were assessed in our multicenter study, including a cohort of 56 IBD patients with incident CRC. The clinical and histopathological features of IBD patients and of CRC were recorded. Incident CRC in IBD occurred at a young age (≤40 years) in 25% of patients (median age 55.5 (22–76)). Mucinous signet-ring carcinoma was detected in 6 out of the 56 (10.7%) patients, including 4 with Ulcerative Colitis (UC) and 2 with Crohn’s disease (CD). CRC was more frequently diagnosed by colonoscopy in UC (85.4% vs. 50%; *p* = 0.01) and by imaging in Crohn’s Disease CD (5.8% vs. 31.8%; *p* = 0.02). At onset, CRC-related symptoms occurred in 29 (51.9%) IBD patients. The time interval from the diagnosis of IBD to CRC was shorter in UC and CD patients with >40 years (*p* = 0.002; *p* = 0.01). CRC-related death occurred in 10 (29.4%) UC and in 6 (27.2%) CD patients (*p* = 0.89), with a short time interval from CRC to death (UC vs. CD: 6.5 (1–68) vs. 14.5 (8–40); *p* = 0.85; IBD: 12 months (1–68)). CRC occurring at a young age, a short time interval from the diagnosis of IBD to CRC-related death in the elderly, CRC-symptoms often mimicking IBD relapse and the observed high mortality rate may support the need of closer surveillance intervals in subgroups of patients.

## 1. Introduction

The risk of developing colorectal cancer (CRC) is increased in Inflammatory Bowel Disease (IBD). Long-standing extensive Ulcerative Colitis (UC), Crohn’s Disease (CD) colitis [1,2,3,4], sclerosing cholangitis [5,6] and chronically active inflammation represent the main risk factors for CRC related to IBD [1,2,3,4]. The sequence “chronic inflammation-dysplasia-cancer” is a well-known pathogenetic mechanism involved in the development of CRC [6,7,8,9,10,11,12,13,14] and also of other cancers involving the gastrointestinal (GI) tract [7,15,16,17]. Diagnosis of CRC at a younger age has been reported in IBD, and surveillance programs have been shown to reduce CRC risk, but not the mortality rate in these patients [2].

CRC risk has been extensively investigated in IBD in terms of frequency, risk factors, the role of immunomodulators [1,2,18,19,20,21,22,23] and endoscopic surveillance, particularly in high-risk patients [24]. However, fewer data are available regarding the long-term outcome of IBD patients with incident CRC.

In a multicenter nested case-control 6-year study [25], we reported the frequency and risk factors for any incident cancer in a cohort of IBD patients. In order to provide additional data regarding the long-term outcome of IBD patients with incident CRC, this issue was addressed in the present study. A subgroup of patients from the previous cohort [25], followed-up during a longer period, was considered for this purpose.

The primary end-point was to assess, in a multicenter study, the long-term outcome of UC and CD patients with incident CRC, in relation to the characteristics of both the patients and CRC. The secondary aim was to evaluate the features of UC and CD patients with CRC, including cancer-related symptoms and diagnostic modalities, in relation to the outcome. Therefore, previously unreported data regarding incident CRC occurring in a subgroup of UC and CD patients from our previous cohort [25], followed-up during a longer period, were analyzed in relation to the outcome.

## 2. Materials and Methods

The frequency and overall risk of any incident cancer occurring from 2011 to 2017 in patients with IBD was previously reported in our multicenter IG-IBD study [25]. In the original cohort [25], the inclusion criteria were a well-defined diagnosis of UC or CD ≥ 6 months [26,27], regular follow-up (≥2 visits/year), age ≥ 17 years, no history of neoplasia, data available in clinical records. Patients with an unclassified or indeterminate IBD or low compliance (<2 visits/year at the referral center) were excluded. In our previous study [25], each IBD patient with cancer was matched for the clinical variables (gender, age ± 5 years, IBD type), with 2 IBD controls with no cancer. In the present study, only IBD patients with incident CRC with no controls were considered. In our previous study [25], no details were reported regarding the clinical outcome and characteristics of CRC (site, histotype, history of adenomas, cancer treatment, mortality) [25]. CRC-related symptoms and the modality of diagnosis of CRC were also not reported [25]. Due to the relatively limited data regarding the characteristics of incident CRC in relation to the long-term outcome of patients with IBD, these issues were specifically investigated.

### 2.1. Study Population

The study population included a subgroup of IBD patients from the previous cohort [25], fulfilling the inclusion and exclusion criteria reported in Table 1. In each center, the available and previously unreported characteristics of CRC and data regarding the clinical outcome of patients followed-up after the end of our previous study (31 December 2017) [25] were retrospectively reviewed until 31 December 2020.

For each patient, detailed demographic and clinical characteristics, including IBD type (UC vs. CD) and extent [26,27], CD phenotype [27], history of surgery and perianal (PA) disease and IBD-related treatments at enrollment were already reported in a database [25].

The following additional characteristics of the CRC and clinical outcome of IBD patients with incident CRC were added in a new database for the purpose of the present study: date of the first and last visit (year) after the end of our previous 6-year study [25], age at diagnosis of CRC, CRC histotype, CRC site (rectum, sigmoid colon, descending colon, transverse colon, right colon, coecum, ileo-cecal valve, anal canal), date at diagnosis of CRC (year), symptoms related to CRC at diagnosis (yes/no, type), modality of diagnosis of CRC (colonoscopy, imaging, intra-operatory: yes/no), concomitant colonic adenomas at diagnosis of CRC (yes/no), history of colonic adenoma (yes/no), family history of CRC, surgery for CRC (yes/no, type), endoscopic treatments for CRC (endoscopic mucosal resection EMR, endoscopic submucosal dissection, ESD), CRC-related death (yes/no, date), death not related to CRC (yes/no, date, cause), lost to follow-up (yes/no), date at the last visit (year), follow-up duration after the diagnosis of CRC (months). CRC-related death was defined as a death due to cancer-related complications or to cancer progression. The diagnosis of CRC was histologically confirmed and defined in all patients by biopsy specimens taken during colonoscopy or surgery. The CRC site was defined during either surgery or colonoscopy/imaging in patients not surgically treated. In each patient with incident CRC, the use of TNF-αantagonists (≥1 administration) and/or other immunomodulators (≥3 months) for IBD was considered only after the diagnosis of CRC.

The study protocol was approved by the ethic committees (referral center: Policlinico “Tor Vergata” of Rome, Italy; 56/15, amendment 15 July 2020). All authors made the decision to submit the manuscript for publication and approved the submitted manuscript. Patient consent was waived as the reported data regarding CRC were retrospectively collected, the research involved no risk for participants and all data were deidentified.

### 2.2. Statistical Analysis

The results were expressed as median (range). The differences between CD and UC patients were analyzed by the χ^2^-square test or by the two-tailed Student’s *t*-test, as appropriate. For this purpose, the following parameters were considered: median age at diagnosis of incident CRC, IBD duration at diagnosis of CRC, CRC site, frequency of CRC-related death, time interval from CRC diagnosis to CRC-related or unrelated death, family history of CRC and history of adenomas, frequency of events (i.e., death).

Regarding the sample size, the study is a post-hoc analysis of our previous prospective study including a subgroup of IBD patients with incident cancer occurring from 2011 to 2017 (25), with an available long-term follow-up. Due to the retrospective study design of this post-hoc analysis, no sample size calculation was performed. Nevertheless, the following probabilistic considerations were taken into account when comparing UC vs. CD groups: (a) The null hypothesis is rejected if the sample mean (of survival times) differs by ≥43.3% of the standard deviation from the corresponding mean of the reference population; (b) To test the hypotheses (null and alternative), the two-tailed Student’s *t*-test was adopted with α = 0.05 (first type error) and β = 0.10 (second type error) (test power equal to 90%). Considering these probabilistic considerations, the number of 56 patients enrolled in the study appears to be congruous.

A Kaplan–Meier analysis was performed in order to assess the survival from the diagnosis of cancer or of IBD to death in different subgroups of IBD patients. The same analysis was used in order to assess the time intervals from the diagnosis of IBD and CRC or death, when considering comparisons between the UC and CD subgroups. The statistical test used for Kaplan–Meier survival was the Log Rank (Mantel Cox). A *p* < 0.05 level was considered for statistical significance.

## 3. Results

### 3.1. Study Population

Overall, 56 patients with IBD and incident CRC fulfilled the inclusion criteria, as details regarding the long term outcome were not available for the remaining 37 patients from the original cohort [25]. The demographic and clinical characteristics of the whole group of 56 IBD patients and, separately, of the 22 CD and 34 UC patients with incident CRC are summarized in Table 2. In 25% (10 out of 56) of the IBD patients, CRC occurred before the age of 40 and in 35.7% (20 out of 36) before the age of 50 years (median: 55.5 (22–76); UC 59 (22–76) vs. CD 53 (32–76); *p* = 0.46) (Table 2). A low frequency of family history of CRC, a personal history of adenomas and comorbidities were observed (Table 2). The median length of the follow-up of IBD patients with CRC, including the time interval between the diagnosis of CRC (enrollment) and the last visit or death was 16.5 months.

The IBD-related lesions in the 22 CD patients with incident CRC included: ileum (*n* = 7; 31.8%), colon (*n* = 4; 18.2%), ileum-colon (*n* = 10; 45.5%), upper GI (*n* = 1; 4.5%) (Table 2). The CD phenotype was non-fibrostricturing non-perforating (B1) in 10 (45.5%), fibrostricturing (B2) in 5 (22.7%) and perforating (B3) in 7 (31.8%) patients (Table 2). Among the 34 UC patients with incident CRC, the disease was left-sided in 13 (38.2%) and extensive in 21 (61.8%), with no cases of proctitis (Table 2).

When comparing the UC and CD groups, no significant differences were observed in terms of gender, median age at diagnosis of CRC and IBD duration at diagnosis of CRC (*p* = 0.13; *p* = 0.46 and *p* = 0.97, respectively) (Table 2). The tested UC and CD populations were also comparable in terms of the time interval from the diagnosis of CRC to the last visit or death (*p* = 0.6) or to death (*p* = 0.85) and from the diagnosis of UC or CD to death (*p* = 0.89) (Table 2).

**Figure 1:**
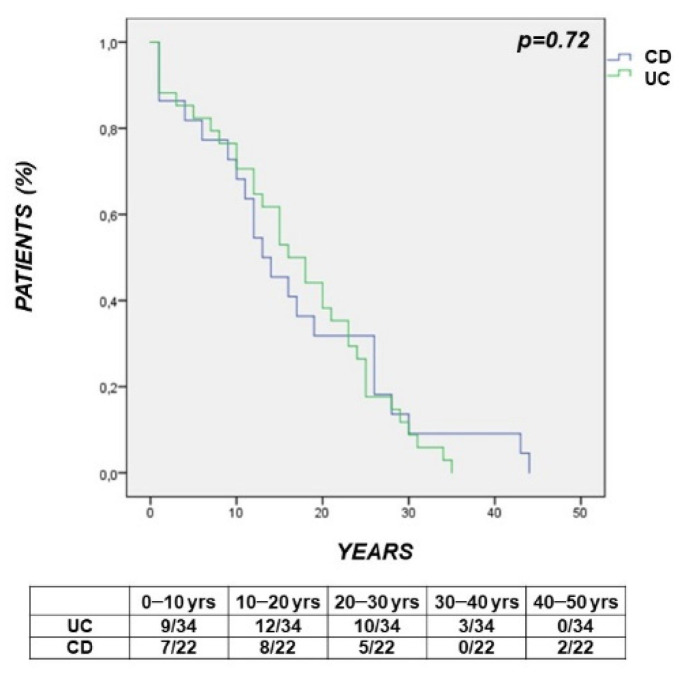
Survival curve showing that the time interval between the diagnosis of Inflammatory Bowel Disease (IBD) and the diagnosis of colorectal cancer (CRC) was comparable in Ulcerative Colitis (UC) vs. Crohn’s Disease (*p* = 0.72).

The survival curve showed that the time interval between the diagnosis of IBD and the diagnosis of CRC was comparable in UC vs. CD patients with incident cancer (*p* = 0.72) (Figure 1).

As expected, a present/past history of IBD-related surgery was more frequent in the tested CD vs. UC population (*p* = 0.002) (Table 2). A history of >1 IBD-related surgery was recorded in 2 of the 9 CD patients (*n* = 2 in 1; *n* = 3 in 1) and in no UC patients. The proportion of patients with a history of smoking, appendectomy or extraintestinal manifestations did not differ between UC and CD groups (*p* = 0.98; *p* = 0.89 and *p* = 0.83, respectively) (Table 2). A comparably low frequency of well-defined family history of CRC and personal history of adenomas or comorbidities was observed in UC vs. CD patients with CRC (*p* = 0.6; *p* = 0.83; *p* = 0.55, respectively) (Table 2).

### 3.2. Characteristics of Incident CRC

#### 3.2.1. CRC Site

In the cohort of 56 IBD patients, incident CRC mostly involved the rectum. In particular, the CRC site included: rectum in 24 (42.8%), sigmoid colon in 10 (17.9%), left colon in 5 (8.9%), transverse colon in 4 (7.1%), right colon in 8 (14.3%), cecum in 2 (3.6%), ileo-cecal valve in 2 (3.6%) and anal canal in 1 (1.8%) patient. When subgrouping the CRC localization into the left-sided (rectum, sigmoid, left colon) vs. right-sided colon (ileo-cecal valve, coecum, ascending, transversus colon), CRC was more frequently left-sided vs. right-sided (40 (71.4%) vs. 16 (28.6%); *p* < 0.0001). The distribution of the CRC site was comparable between UC and CD (Table 3). CRC occurred in areas involved by UC lesions in 31 out of the 34 patients, while in CD patients CRC occurred in disease-uninvolved areas in the 7 patients with ileal CD. In the overall IBD population, a significantly longer time interval from the diagnosis of IBD to the diagnosis of CRC (IBD duration) was observed in patients with rectal vs. other colonic cancers (23 (1–44) vs. 12 (0–43) years, *p* = 0.01). Differently, the median age at diagnosis of CRC did not differ between IBD patients with rectal vs. other colonic cancers (54 (23–75) vs. 55.5 (22–76) years; *p* = 0.51). Neither was any difference observed between UC and CD patients in terms of the median age at diagnosis of rectal cancer (54 (23–75) vs. 53 (33–66) years; *p* = 0.61), nor in terms of the time interval from the diagnosis of IBD to rectal cancer (23 (1–35) vs. 26 (10–44) years; *p* = 0.44).

#### 3.2.2. CRC Histotype

The characteristics of CRC are summarized in Table 3. In the overall IBD population (*n* = 56), the CRC histotype was mostly represented by adenocarcinoma (*n* = 51 (91%)). Among the IBD patients with incident adenocarcinoma, there were 6 mucinous signet-ring carcinoma (4 UC, 2 CD), leading to death in 2 patients. The remaining 5 CRC included neuroendocrine tumor (*n* = 2 (3.6%)), squamous carcinoma (*n* = 1 (1.8%)), and epidermoid carcinoma (*n* = 1 (1.8%)), while the histology was undefined in 1 (1.8%) case (Table 3).

The frequency of each CRC histotype did not significantly differ between the UC and CD groups (Table 3).

#### 3.2.3. CRC-Related Symptoms

Symptoms suggesting a diagnosis of CRC were reported in only 29 out of the 56 (51.9%) IBD patients with incident cancer (Table 3). In IBD patients, the CRC-related symptoms at the diagnosis of cancer included rectal bleeding in 13 (23.6%), abdominal pain in 10 (17.8%), refractory IBD-related symptoms in 5 (8.9%), diarrhea in 4 (7.1%), occlusion in 3 (5.3%) and changes in bowel habits in 3 (5.3%) patients (Table 3). Overall, a comparable proportion of UC and CD patients showed symptoms leading to a diagnosis of CRC (*p* = 0.62) (Table 3). The same was observed when separately considering each symptom (Table 3).

#### 3.2.4. Diagnosis of CRC: Modalities

In the tested IBD population, CRC was diagnosed by colonoscopy in 40 patients (71.4%) and by imaging in 9 (16.1%), while the diagnosis was intra-operatory in 7 (12.5%) patients (Table 3).

When comparing UC and CD, CRC was more frequently diagnosed by colonoscopy in UC (29 (85.4%) vs. 11 (50%); *p* = 0.01) and by imaging in CD (2 (5.8%) vs. 7 (31.8%); *p* = 0.02) (Table 3). In a comparable low proportion of UC and CD patients, an intra-operatory diagnosis of CRC was made (*p* = 0.53) (Table 3). In both UC and CD patients the time interval between the diagnosis of CRC and CRC-related death was comparable between patients with a diagnosis of cancer made by colonoscopy vs. other techniques (imaging or intra-operatory) (*p* = 0.58 and *p* = 0.54, respectively).

### 3.3. Current or Past History of Adenomas

At the time of the diagnosis of CRC, concomitant colonic adenomas were reported in 4 (7.1%) IBD patients, all with UC (4 (11.7%) vs. 0 in CD; *p* = 0.25) (Table 3). The history of endoscopic removal of adenomas before the occurrence of CRC was recorded in an additional 7 (12.5%) IBD patients (UC vs. CD: 4 (11.7%) vs. 3 (13.6%); *p* = 0.83) (Table 2).

#### 3.3.1. CRC: Surgical or Endoscopic Treatment

The CRC treatment included surgical or endoscopic treatment in 51 (91.1%) IBD patients (UC vs. CD: 30 (88.2%) vs. 21 (95.5%); *p* = 0.65) (Table 3). Specifically, endoscopic or surgical CRC treatment included: palliative stoma in 3 (5.9%), endoscopic removal in 3 (5.9%), anterior rectal resection in 1 (1.9%), ileo-colonic resection in 1 (1.9%), hemicolectomy in 6 (11.8%), colectomy with colostomy in 3 (5.9%), colectomy with ileostomy in 16 (31.4%), ileal pouch in 9 (17.6%) and ileo-rectal anastomosis in 9 (17.6%) patients.

Among the 51 surgical procedures for CRC in IBD patients, hemicolectomy was more frequent in CD vs. UC (6 (28.6%) vs. 0 (0%); *p* = 0.006), and ileal pouch in UC vs. CD (9 (30%) vs. 0 (0%); *p* = 0.02) (Table 3). The frequency of other surgical treatments for CRC did not differ between the two groups (Table 3).

#### 3.3.2. Characteristics of IBD

**Figure 2:**
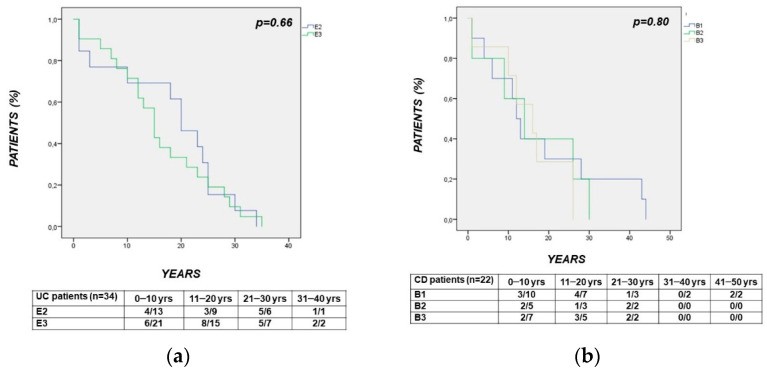

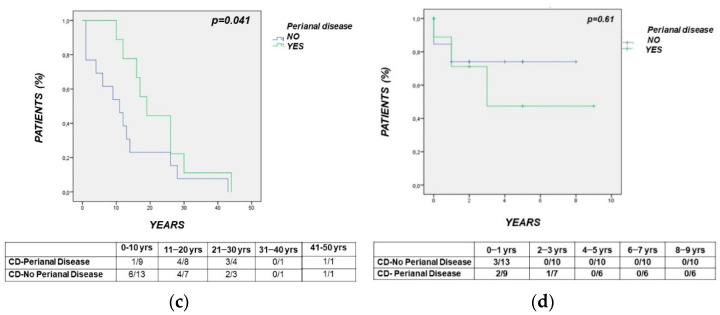
(panels **a**–**d**): Survival curves showing the time interval from: panel (**a**). Diagnosis of UC to diagnosis of CRC, comparable between patients with left-sided vs. extensive UC (E2 vs. E3: *p* = 0.66); panel (**b**). Diagnosis of CD to diagnosis of CRC, comparable between patients with different CD behaviors (B1 vs. B2 vs. B3: *p* = 0.80); panel (**c**). Diagnosis of CD to diagnosis of CRC, significantly longer in patients with vs. without perianal (PA) disease (*p* = 0.04); panel (**d**). Diagnosis of CRC to CRC-related death, comparable between CD patients with vs. without PA disease (*p* = 0.61).

The time interval from the diagnosis of IBD to the diagnosis of CRC was comparable when in patients with left-sided vs. extensive UC (E2 vs. E3: *p* = 0.66) (Figure 2a) or with different CD behaviors (B1 vs. B2 vs. B3: *p* = 0.80) (Figure 2b). In CD, the time interval between the diagnosis of CD and the diagnosis of CRC was longer in patients with vs. without perianal (PA) disease (*p* = 0.04) (Figure 2c). The observed lower median age in CD patients with vs. without perianal disease was at the limit of statistical significance (years: 48 (22–66) vs. 58 (34–76); *p* = 0.05). The time interval between the diagnosis of CRC and CRC-related death was comparable between CD patients with vs. without PA disease (*p* = 0.61) (Figure 2d).

**Figure 3:**
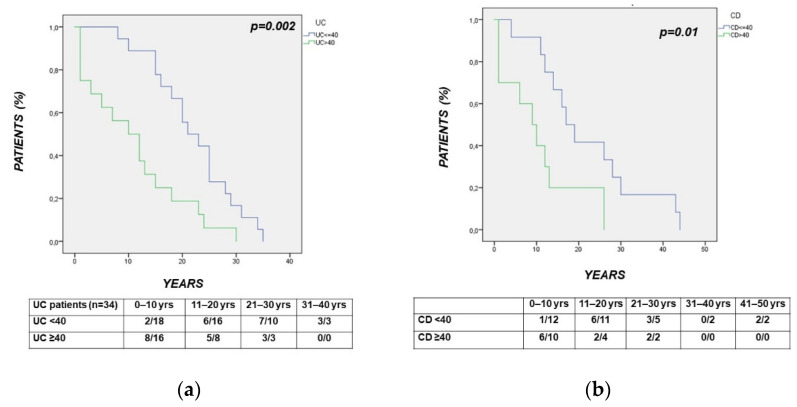
(panels **a**,**b**) Kaplan–Meier analysis showing that the time interval between the diagnosis of IBD and the diagnosis of CRC was significantly shorter in patients with IBD diagnosed with >40 vs. ≤40 years, both in UC (*p* = 0.002) (panel **a**) and in CD (*p* = 0.01) (panel **b**).

In UC, the Kaplan–Meier analysis showed that the time interval between the diagnosis of UC and the diagnosis of CRC was significantly shorter in patients with UC diagnosed >40 vs. ≤40 years (*p* = 0.002) (Figure 3a). As for UC, the time interval between the diagnosis of CD and the diagnosis of CRC was shorter in CD patients diagnosed with >40 vs. ≤40 years (*p* = 0.01) (Figure 3b).

### 3.4. Immunomodulatory Treatments

In the whole group of 56 IBD patients, a current or previous history of conventional immunomodulators (ISS) or biologics was recorded in 19 (33.9%) and in 18 (32.1%) patients, respectively (Table 2). A comparable frequency of ISS or biologics use was observed in the tested UC vs. CD populations with incident CRC (Table 2).

The time interval from the diagnosis of IBD to the diagnosis of CRC was comparable between UC vs. CD patients using (*p* = 0.40) or not using biologics (*p* = 0.88). No significant differences were observed in terms of the time interval from the diagnosis of IBD to CRC when comparing UC vs. CD patients using (*p* = 0.66) or not using ISS (*p* = 0.99). The time interval between diagnosis of CRC and CRC-related death did not differ between UC or CD patients using (*p* = 0.32) vs. not using biologics (*p* = 0.70).

In the UC group, the time interval from the diagnosis of CRC and CRC-related death did not differ between patients with vs. without history of ISS use (*p* = 0.67). In the tested CD population, this difference was at the limit of statistical significance in the subgroup of CD patients using ISS (*p* = 0.045). The median age of these 2 groups of CD patients was comparable (ISS vs. no ISS: 54.5 (22–70) vs. 58.5 (23–76); *p* = 0.21). Among the 8 CD patients using ISS, 5 showed a history of perianal disease and 5 of biologics use. These characteristics were observed in a minor proportion of CD patients not using ISS (5 out of 14 for both perianal disease and biologics use).

### 3.5. Death

Overall, death related or not to CRC occurred in 18 (32.1%) IBD patients, at a comparable frequency in UC vs. CD (10 (29.4%) vs. 8 (36.4%); *p* = 0.8) (Table 3). CRC-related death occurred in 16 (28.5%) IBD patients, with no differences between UC and CD (10 (29.4%) vs. 6 (27.2%); *p* = 0.89).

**Figure 4:**
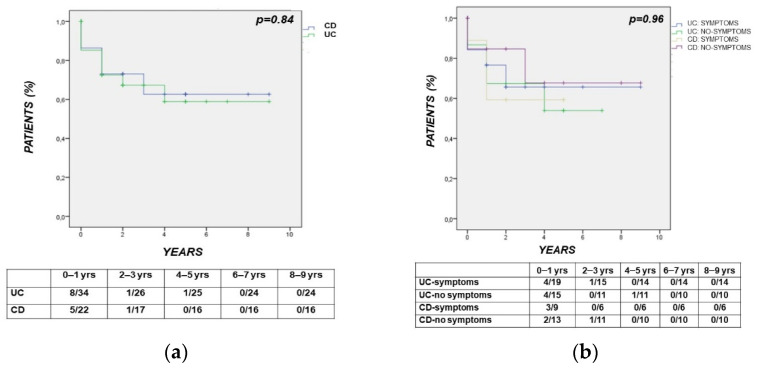
(panels (**a**,**b**)) Kaplan–Meier analysis showing that the time interval between the diagnosis of CRC and CRC death did not differ between UC and CD (*p* = 0.84) (panel (**a**)) and between IBD patients subgrouped according to a positive vs. negative history of symptoms leading to a diagnosis of CRC (*p* = 0.96) (panel (**b**)).

In IBD, the median time interval between the diagnosis of CRC to death was 12 months (1–68), in particular 6.5 (1–68) in UC vs. 14.5 (8–40) months in CD (*p* = 0.85). According to the survival analysis, the time interval between the diagnosis of CRC to CRC-related death did not differ between UC and CD (*p* = 0.84) (Figure 4a). The same time interval (diagnosis of CRC to CRC-related death) was also comparable between IBD patients subgrouped according to a positive vs. negative history of symptoms leading to a diagnosis of CRC (*p* = 0.96) (Figure 4b).

## 4. Discussion

CRC risk is increased in patients with a long-term history of IBD colitis [1,2,3,4,5,6,7]. Several risk factors for CRC, including those common to the general non-IBD population, have been reported in IBD [1,2]. However, fewer studies evaluated the clinical outcome of IBD patients with incident CRC in relation to the characteristics of both the host and the cancer. Additional data may help to optimize the CRC surveillance and treatment in IBD patients with concomitant cancer. On the basis of these observations, the long-term outcome of incident CRC in IBD patients was investigated in relation to features of IBD and cancer. The cases included a subgroup of IBD patients with incident CRC from a 6-year study [25], followed-up during a longer period. Previously unreported features of CRC and IBD patients have been firstly reported in the present study and related to the clinical outcome.

The demographic and clinical characteristics observed in UC and CD patients with CRC, including the age at diagnosis of IBD and the frequency of PA disease, are in agreement with the current literature [1,2,6,11]. This supports the reliability of our findings, as bias related to a selected IBD subpopulation should be excluded. A wide range in terms of age at diagnosis of CRC was observed (22–76 years). CRC indeed occurred before the age of 40 in almost one fourth of patients, and before the age of 50 in more than one third of patients. Current evidence supports a younger age at diagnosis of CRC in IBD patients compared to the general non-IBD population [2,26,28,29,30]. The observed trend for a higher frequency of CRC in males and the low proportion of young IBD patients with CRC and long-standing colitis was also expected [31]. This last finding further supports the need to search for additional risk factors for CRC in young IBD patients, in order to optimize the surveillance programs in these patients.

The reliability of the tested IBD population is also supported by the expected observed comparable time interval from the diagnosis of IBD to incident CRC in the UC vs. CD groups [2]. A longer IBD duration was observed in patients with rectal vs. other colonic cancers, thus suggesting a role for a long-lasting rectal inflammation in the development of CRC. In our UC population, no cases of proctitis were observed, according to the well-known higher CRC risk in patients with left-sided or extensive colitis, but not with proctitis [1,2,3,6,7,31]. This observation also suggests that more severe cases of UC were included in our study, as expected in tertiary IBD referral centers.

The colonic cancer distribution was also expected, as CRC involved the rectum, sigmoid or transversus colon in more than half of IBD patients and in two/thirds of UC patients. Sporadic cancer was observed in 3 out of the 13 UC patients with left-sided colitis. These findings are in agreement with the data from both the IBD and non-IBD populations [2,4,5,6]. Interestingly, symptoms compatible with CRC were observed in a relatively low proportion of patients (half of our IBD population), while the remaining patients showed CRC-related symptoms compatible with IBD relapse. This observation further supports that a timely diagnosis of CRC in IBD mostly relies on appropriate screening programs rather than on searching for CRC-related symptoms, most often mimicking IBD relapse.

The diagnostic modalities for detecting CRC differed between our CD vs. UC populations. Due to the retrospective design, CRC surveillance intervals were not set, thus representing one of the limitations of the study. However, as all patients referred to tertiary IBD centers, a CRC surveillance according to the current IBD guidelines [26,27] was reasonably applied. CT scan and other imaging techniques rather than colonoscopy were performed in relation to disease-related clinical indications and not for CRC screening. In our cohort, colonoscopy was the technique most frequently leading to a diagnosis of CRC in the UC group, while, surprisingly, imaging allowed more often for a diagnosis of CRC in CD. This observation suggests that even in tertiary IBD referral centers, endoscopic screening may not always enable a timely diagnosis of CRC, particularly in CD. However, the retrospective study design did not allow for comparisons in terms of TNM staging at diagnosis. Due to this finding, a diagnostic delay leading to a higher mortality for CRC in the tested CD vs. UC population was conceivable. However, this difference was not significant in our IBD population, in agreement with current evidence indicating a comparable mortality rate for CRC in CD vs. UC [32]. Although CRC is more frequently diagnosed by imaging in CD, the time interval from the diagnosis of CRC to CRC-related death was comparable in UC versus CD.

Among the more relevant findings, the time interval from the diagnosis of UC or CD to the diagnosis of CRC was shorter in patients with IBD diagnosed with >40 vs. ≤40 years. This cut-off was considered, as it is used for subgrouping IBD patients in relation to the age at onset (Montreal classification, A2: <40 years) [33]. The observed difference showed a high level of significance in both UC and CD (*p* < 0.002 and *p* < 0.01, respectively). Delayed surveillance programs in elderly IBD patients may be involved in this finding, as well as the well-established higher CRC risk in the elderly [31]. A delayed diagnosis of IBD determining an inappropriate CRC surveillance before the diagnosis of UC or CD may also be hypothesized. Present findings may suggest the need for earlier surveillance programs in the elderly and in the late-diagnosed IBD.

Perianal disease has recently been reported to increase CRC risk in CD [34]. In our population, the presence of perianal disease did not influence the time interval from the diagnosis of CRC to CRC-related death. However, a longer time interval from the diagnosis of CD to CRC was observed in patients with perianal disease. The observed trend at the limit of the statistical significance for a lower median age at diagnosis of CRC in patients with vs. without perianal disease (*p* = 0.05) may be involved in this finding. A closer endoscopic surveillance in CD patients with perianal disease aimed to assess the activity of rectal lesions may be involved in our finding. Severe rectal lesions have indeed been associated with a worse course of perianal disease [27].

Whether biologics may increase the cancer risk has been investigated in IBD [2,18,19,20,21,22,23,25,35,36]. This is particularly true for TNF-α antagonists, as this cytokine induces the necrosis of cancer cells in vitro [37]. In our cohort, biologics appeared not to influence the time interval from the diagnosis of IBD to CRC and from the diagnosis of CRC to CRC-related death. ISS did not affect the time interval from the diagnosis of IBD to CRC, while a shorter time interval at the limit of statistical significance (*p* = 0.045) from the diagnosis of CRC to CRC-related death was observed in CD patients using ISS. However, the observed number of cases (history of ISS and CRC in CD) was very limited (*n* = 8). Moreover, this small subgroup of patients using ISS also showed a more severe CD course (PA disease and/or biologic use in 5 out of the 8 patients), while an older age at diagnosis of CRC appeared not to be involved in this finding. A large French study reported a lower frequency of CRC in IBD patients with severe colitis using ISS [24], while a Danish Nationwide study (1997–2015) subsequently reported that medical treatment does not affect the CRC risk in CD [38].

As expected, adenocarcinoma was the more frequent CRC histotype in our UC and CD populations [1,2]. However, colonic neuroendocrine tumors were also observed (3.6%), as occasionally reported in IBD [39]. A relatively high proportion of incident mucinous signet ring carcinoma-type occurred in the tested IBD population, as rarely reported [40]. A present or past history of colonic adenomas was observed in a low proportion of UC and CD patients (about one tenth), suggesting that the tested population was not at increased risk of developing CRC not related to IBD. Among the limitations of our study, the family history of CRC was not available for all patients.

The frequency of CRC-related death was quite high, being observed in almost two/thirds of patients (28%), with no differences between CD and UC. A long time interval from the diagnosis of CRC to death was observed in both UC and CD patients, including within the first year of disease, in agreement with previous studies [41].

The diagnostic techniques most frequently leading to a diagnosis of CRC included colonoscopy in UC and other modalities in CD, representing one of our more relevant findings. This observation suggests that even when considering IBD referral centers, an unexpected diagnosis of CRC can be made by imaging or during surgery, particularly in CD. Features of CRC-related symptoms in IBD, which may mimic disease relapse, as observed in our population, may play a role in this finding. CRC involved the left colon in almost two/thirds of the IBD patients. However, among the initial CRC-related symptoms, rectal bleeding occurred in less than one fourth of patients or was compatible with disease relapse. Other symptoms leading to a CRC diagnosis were also compatible with an IBD relapse, particularly in CD. These findings suggest the need to carefully search for a possible concomitant colonic neoplasia in UC and CD patients developing a refractory IBD course.

Among the limitations of the study there is the relatively small cohort of IBD patients considered in the analysis. No sample size calculation was performed, in relation to our retrospective post-hoc analysis and to the limited data regarding the long-term outcome of IBD patients with incident CRC related to the characteristics of the patients and the cancer. Nevertheless, according to the previously reported probabilistic considerations, the number of CD and UC patients considered appeared congruous. The present findings may help to calculate the sample size for future studies addressing this issue in IBD.

The strength of our investigation includes the multicenter study assessing the clinical characteristics and outcome of incident cases of CRC in IBD patients followed-up in a long term. The homogeneous cohort of IBD patients, with detailed demographic and clinical characteristics, should also allow for adequate comparisons between UC and CD patients without bias related to inadequate sampling [26,27].

## 5. Conclusions

In conclusion, among the major findings of the present study, there is the observed shorter time interval between the diagnosis of either UC or CD and the diagnosis of CRC in older patients, suggesting a delayed surveillance in elderly IBD patients. The present findings may suggest the need to reconsider the timing of endoscopic surveillance in elderly patients with IBD. The observed occurrence of CRC also in young IBD patients strongly supports the need to search for additional risk factors for cancer and to optimize the surveillance programs in this subpopulation. Overall, our multicenter study may provide additional clinically useful information regarding the symptoms at the onset, natural history and the outcome of incident CRC in IBD patients under a regular follow-up.

## Figures and Tables

**Table 1:** Inclusion and exclusion criteria.

Inclusion Criteria	Exclusion Criteria
IBD patients with incident CRC occurring during our 6-year study [25] with additional data not previously reported	Incomplete data regarding the CRC characteristics and/or clinical outcome of IBD patients with incident CRC after the end of the first study (31 December 2017)
Diagnosis of CD or UC according to current European guidelines [26,27]	Patients from the previous cohort [25] with incident cancer different from CRC
Well-defined diagnosis of incident CRC (31 December 2011–31 December 2017) [25]	All IBD controls with no cancer included in our previous study [25]
Detailed clinical data regarding IBD and CRC, including the clinical outcome of CRC after the end of our first study (31 December 2017) [25]	Low compliance (<2 visits/year)
Diagnosis of IBD lasting ≥6 months	
Regular follow up (≥2 visits/year)	
Age ≥ 17 years	
Well-defined characteristics of CRC including: Histotype, Site, CRC treatment (surgery, chemo/radiotherapy), Diagnostic modalities of CRC, Personal history of adenomas	
Well-defined characteristics of CRC including: Histotype, Site, CRC treatment (surgery, chemo/radiotherapy), Diagnostic modalities of CRC, Personal history of adenomas	

Abbreviations. IBD: Inflammatory Bowel Disease; UC: Ulcerative Colitis; CD: Crohn’s Disease; CRC: colorectal cancer.

**Table 2:** Demographic and clinical characteristics of IBD patients with incident CRC.

Kerrypnx	Total (*n* = 56)	UC (*n* = 34)	CD (*n* = 22)	p (Yates)
Gender (F) *n* (%)	20 (35.7%)	9 (26.4%) ^a^	11 (50%)	0.13
Age at CRC diagnosis, median (range)	55.5 (22–76)	59 (22–76)	53 (32–76)	0.46
IBD duration at CRC, median (range)	15.5 (1–44)	17 (1–35)	13.5 (1–44)	0.97
IBD related surgery, *n* (%)	11 (19.6%)	2 (5.9%)	9 (40.9%)	0.002
Time interval from diagnosis of IBD to last visit/death (yrs), median (range) (*n* = 50)	1 (0–21)	1 (0–9)	1 (0–21)	0.37
Time interval from diagnosis of IBD to CRC-related death (years), mean (range) (*n* = 16)	15.5 (1–45)	18.5 (1–28)	12.5 (2–45)	0.89
Time interval from diagnosis of CRC and CRC-related death (months), mean (range) (*n* = 16)	12 (1–68)	6.5 (1–68)	14.5 (8–40)	0.85
Time interval between CRC diagnosis and death/loss at follow up (years), mean (range)	2 (0–41)	2 (0–41)	1.5 (0–21)	0.60
Perianal disease, *n* (%)	11 (19.6%)	2 (5.9%)	9 (40.9%)	0.004
Smoking (yes), *n* (%)	4 (7.1%)	2 (5.8%)	2 (9.1%)	0.98
Appendectomy, *n* (%)	6 (10.7%)	4 (11.7%)	2 (9.1%)	0.89
EIMs, *n* (%)	7 (12.5%)	4 (11.7%)	3 (13.6%)	0.83
Comorbidities, *n* (%)	24 (42.8%)	13 (38.2%)	11 (50%)	0.55
Family history CRC, *n* (%)				0.6
yes	5 (8.9%)	2 (5.8%)	3 (13.6%)	
no	46 (82.2%)	27 (79.4%)	19 (86.4%)	
unknown	5 (8.9%)	5 (14.7%)	0 (0%)	
History of adenomas, *n* (%)	7 (12.5%)	4 (11.7%)	3 (13.6%)	0.83
CD behavior, *n* (%)				
B1	-	-	10 (45.5%)	
B2	-	-	5 (22,7%)	
B3	-	-	7 (31.8%)	
CD location, *n* (%)				
L1	-	-	7 (31.8%)	
L2	-	-	4 (18.2%)	
L3	-	-	10 45.5%)	
L4	-	-	1 (4.5%)	
UC extent, *n* (%)				
E1	-	0 (0%)	-	
E2	-	13 (38.2%)	-	
E3	-	21 (61.8%)	-	
Previous or ongoing ISS, *n* (%)	19 (33.9%)	11 (32.3%)	8 (36.4%)	0.98
Thiopurines	19 (33.9%)	11 (32.3%)	8 (36.4%)	0.98
Methotrexate	2 (3.5%)	0 (0%)	2 (9.1%)	0.29
Previous or ongoing biologics, *n* (%)	18 (32.1%)	8 (22.2%)	10 (45.5%)	0.15
Infliximab	14 (25%)	6 (16.6%)	8 (36.4%)	0.20
Adalimumab	6 (10.7%)	2 (5.5%)	4 (18.2%)	0.31
Certolizumab	1 (1.8%)	0 (0%)	1 (4.5%)	0.82

Abbreviations. IBD: Inflammatory Bowel Disease; UC: Ulcerative Colitis; CD: Crohn’s Disease; CRC: colorectal cancer; EIMs: Extraintestinal manifestations; B1: non-penetrating non-stricturing, B2: stricturing, B3: penetrating; L1: ileal, L2: colonic, L3 ileum-colonic, L4, upper gastro-intestinal tract; E1: proctitis, E2: left-sided colitis, E3: extended colitis; ISS: conventional immunosuppressors.

**Table 3:** Characteristics and outcome of incident colorectal cancer in the tested IBD population.

	Total (*n* = 56)	UC (*n* = 34)	CD (*n* = 22)	p (Yates)
**CRC Site, *n* (%)**				
Rectum	24 (42.8%)	15 (44.2%)	9 (40.9%)	0.96
Sigmoid colon	10 (17.9%)	8 (23.7%)	2 (9.1%)	0.30
Left colon	5 (8.9%)	4 (11.7%)	1 (4.5%)	0.65
Transverse colon	4 (7.1%)	4 (11.7%)	0 (0%)	0.25
Right colon	8 (14.3%)	2 (5.8%)	6 (27.4%)	0.06
Cecum	2 (3.6%)	1 (2.9%)	1 (4.5%)	0.67
Ileo-cecal valve	2 (3.6%)	0 (0%)	2 (9.1%)	0.29
Anal canal/rectal edge	1 (1.8%)	0 (0%)	1 (4.5%)	0.82
**Left vs. Right Colon, *n* (%)**				
Left Colon	40 (71.4%)	27 (79.4%)	13 (59.1%)	0.17
Right colon	16 (28.6%)	7 (20.6%)	9 (40.9%)	0.17
**CRC Histotype, *n* (%)**				
Adenocarcinoma	51 (91%)	30 (88.4%)	21 (95.5%)	0.65
Squamous carcinoma	1 (1.8%)	1 (2.9%)	0 (0%)	0.82
Epidermoid	1 (1.8%)	1 (2.9%)	0 (0%)	0.82
Neuroendocrine	2 (3.6%)	1 (2.9%)	1 (4.5%)	0.67
N/A	1 (1.8%)	1 (2.9%)	0 (0%)	0.82
**CRC-Related Symptoms, *n* (%)**	29 (51.9%)	19 (55.9%)	10 (45.4%)	0.62
Rectal bleeding	13 (23.6%)	10 (29.4%)	3 (13.6%)	0.29
Abdominal pain	10 (17.8%)	5 (14.7%)	5 (22.7%)	0.68
Refractory IBD	5 (8.9%)	4 (11.7%)	1 (4.5%)	0.65
Diarrhea	4 (7.1%)	1 (2.9%)	3 (13.6%)	0.32
Occlusion	3 (5.3%)	2 (5.8%)	1 (4.5%)	0.69
Change in bowel habits	3 (5.3%)	2 (5.8%)	1 (4.5%)	0.69
**CRC Diagnosis, *n* (%)**				
Colonoscopy	40 (71.4%)	29 (85.4%)	11 (50%)	0.01
Imaging	9 (16.1%)	2 (5.8%)	7 (31.8%)	0.02
Intra-operatory	7 (12.5%)	3 (8.8%)	4 (18.2%)	0.53
**CRC and Concomitant Adenoma, *n* (%)**	4 (7.1%)	4 (11.7%)	0 (0%)	0.25
**Surgical/Endoscopic Treatment, *n* (%)**	51 (91.1%)	30 (88.2%)	21 (95.5%)	0.65
Palliative stoma	3 (5.9%)	3 (10%)	0 (0%)	0.40
Endoscopic removal	3 (5.9%)	1 (3.3%)	2 (9.5%)	0.69
Anterior Rectal Resection	1 (1.9%)	0 (0%)	1 (4.7%)	0.82
Ileo-colonic resection	1 (1.9%)	1 (3.3%)	0 (0%)	0.82
Hemicolectomy	6 (11.8%)	0 (0%)	6 (28.6%)	0.006
Colectomy + colostomy	3 (5.9%)	1 (3.3%)	2 (9.5%)	0.69
Colectomy + ileostomy	16 (31.4%)	8 (26.7%)	8 (38.1%)	0.46
Ileal pouch	9 (17.6%)	9 (30%)	0 (0%)	0.02
Ileo-rectal anastomosis	9 (17.6%)	7 (23.3%)	2 (9.5%)	0.44
**CRC-Related Death, *n* (%)** Yes	16 (28.5%)	10 (29.4%)	6 (27.2%)	0.89
No	36 (64.3%)	22 (64.8%)	14 (63.7%)	
Unknown	4 (7.1%)	2 (5.8%)	2 (9.1%)	
**Non-CRC Related Death, *n* (%)**	2 (3.6%)	0 (0%)	2 (9.1%)	0.29
**Death (Overall), *n* (%)**	18 (32.1%)	10 (29.4%)	8 (36.4%)	0.80
**Lost at Follow-Up, *n* (%)**	20 (35.7%)	13 (38.2%)	7 (31.8%)	0.83

Abbreviations. IBD: Inflammatory Bowel Disease; UC: Ulcerative Colitis; CD: Crohn’s Disease; CRC: colorectal cancer; N/A: not applicable.

## Data Availability

Data available upon request.
